# DNA intercalator BMH-21 inhibits RNA polymerase I independent of DNA damage response

**DOI:** 10.18632/oncotarget.2020

**Published:** 2014-05-26

**Authors:** Laureen Colis, Karita Peltonen, Paul Sirajuddin, Hester Liu, Sara Sanders, Glen Ernst, James C. Barrow, Marikki Laiho

**Affiliations:** ^1^ Department of Radiation Oncology and Molecular Radiation Sciences, Johns Hopkins University School of Medicine, Baltimore, MD 21287, USA; ^2^ Center for Drug Research, University of Helsinki, 00014 Helsinki, Finland; ^3^ Department of Pharmacology, Johns Hopkins University, Baltimore, MD 21205, USA; ^4^ Lieber Institute for Brain Development, Baltimore, MD 21205, USA; ^5^ Sidney Kimmel Comprehensive Cancer Center, Johns Hopkins University School of Medicine, Baltimore, MD 21287, USA

**Keywords:** DNA intercalation, small molecule, DNA damage response, transcription, RNA polymerase I, nucleolus

## Abstract

DNA intercalation is a major therapeutic modality for cancer therapeutic drugs. The therapeutic activity comes at a cost of normal tissue toxicity and genotoxicity. We have recently described a planar heterocyclic small molecule DNA intercalator, BMH-21, that binds ribosomal DNA and inhibits RNA polymerase I (Pol I) transcription. Despite DNA intercalation, BMH-21 does not cause phosphorylation of H2AX, a key biomarker activated in DNA damage stress. Here we assessed whether BMH-21 activity towards expression and localization of Pol I marker proteins depends on DNA damage signaling and repair pathways. We show that BMH-21 effects on the nucleolar stress response were independent of major DNA damage associated PI3-kinase pathways, ATM, ATR and DNA-PK_cs_. However, testing a series of BMH-21 derivatives with alterations in its *N,N*-dimethylaminocarboxamide arm showed that several derivatives had acquired the property to activate ATM- and DNA-PK_cs_ -dependent damage sensing and repair pathways while their ability to cause nucleolar stress and affect cell viability was greatly reduced. The data show that BMH-21 is a chemically unique DNA intercalator that has high bioactivity towards Pol I inhibition without activation or dependence of DNA damage stress. The findings also show that interference with DNA and DNA metabolic processes can be exploited therapeutically without causing DNA damage.

## INTRODUCTION

DNA interaction is a well-recognized property for several classes of cancer drugs, which interact with the duplex DNA with three typical binding modalities, namely DNA intercalation, groove binding and covalent interactions [1, 2]. Most current cytotoxic drugs cause DNA strand lesions, inter- or intrastrand crosslinks or formation of DNA adducts leading to strand breaks during replication and transcription [1, 3]. DNA intercalators are typically small molecule planar molecules that intercalate between DNA bases and cause local structural changes in DNA, including unwinding and lengthening of the DNA strand [2, 4]. These events may lead to alterations in DNA metabolism, halter transcription and replication, and result in both therapeutic advantage and normal tissue toxicity [3, 5].

The acute DNA damage response includes activation of phosphoinositide 3-kinase related damage sensor and transducer kinases ataxia-telangiectasia mutated (ATM) and ATM and Rad3-related (ATR), or DNA dependent protein kinase (DNA-PKcs) [6, 7]. Activated ATM/ATR kinases further propagate the damage signal by phosphorylating a number of downstream target proteins that participate in the DNA damage response (DDR) that includes DNA lesion sensing and marking and mediate processes that lead to effective assembly of the DNA repair complexes at the damage site [8]. Most notably, phosphorylation of H2AX subtype on Ser-139 (named as γH2AX), propagates marking of the DNA lesion and facilitates the formation of DNA damage foci [9]. The rapid kinetics of H2AX marking, sensitivity of its detection, and resolution following lesion repair have prompted its wide use as a DNA lesion marker with proposed uses as a biomarker for chemotherapeutic responses [10]. The efficacy and kinetics of repair, and selection of repair pathways depend also on chromatin compaction, and is especially challenging in the heterochromatin environment [11, 12].

We have recently identified a planar tetracyclic small molecule, named as BMH-21 that intercalates into double strand (ds) DNA and has binding preference towards GC-rich DNA sequences [13, 14]. Based on molecular modeling, we have shown that it stacks flatly between GC bases and that its positively charged sidechain potentially interacts with the DNA backbone [14]. BMH-21 had wide cytotoxic activities against human cancer cell lines, and acts in p53-independent manner, widely considered as a mediator of many cytotoxic agents [14]. We identified BMH-21 as a novel agent that inhibits transcription of RNA polymerase I (Pol I) by binding to ribosomal (r) DNA that caused Pol I blockade and degradation of the large catalytic subunit of Pol I, RPA194.

Given that Pol I transcription is a highly compartmentalized process that takes place in the nucleolus, and that the nucleolus is assembled around this transcriptionally active process, the blockade activated by BMH-21 leads also to the dissolution of the nucleolar structure [14]. Transcription stress of the nucleolus is hence reflected by reorganization of nucleolar proteins that participate in Pol I transcription, rRNA processing and ribosome assembly [15-17]. Considering that Pol I transcription is a highly deregulated pathway in cancers, its therapeutic targeting has substantial promise and has been shown to be effective also using another small molecule, CX-5461 [18-20]. Our studies defined a new action modality for BMH-21 in terms of Pol I inhibition and provided proof-of-principle demonstration that Pol I repression and targeting of RPA194 is a feasible anticancer strategy.

In our initial studies we showed that BMH-21 did not activate ATM-dependent pathways responsible for p53 activity, H2AX or KAP1 phosphorylation [13]. This was intriguing noting the DNA intercalation property of BMH-21 and binding to GC-rich DNA [13, 14], properties which are shared by many polyaromatic heterocyclic intercalators. While many cause DNA damage by electrophilic addition, increased reactive oxygen species production, interfacial inhibition of DNA cleaving enzymes, others like chloroquine change chromatin conformation and activate the ATM pathway [1, 21]. Here we show that BMH-21 activity towards Pol I is independent of DNA damage signaling or repair pathways. We further assessed whether chemical changes introduced to BMH-21 could activate DDR. We show that several derivative molecules, with changes in the BMH-21 basic sidechain, had greatly decreased potencies to inhibit Pol I but caused activation of the DDR response. These findings show that efficient Pol I targeting by the tetracyclic DNA intercalator occurs independent of the DNA damaging activity associated with common intercalators.

## RESULTS

### BMH-21 regulation of RNA Pol I is independent of DNA damage signaling

**Figure 1:**
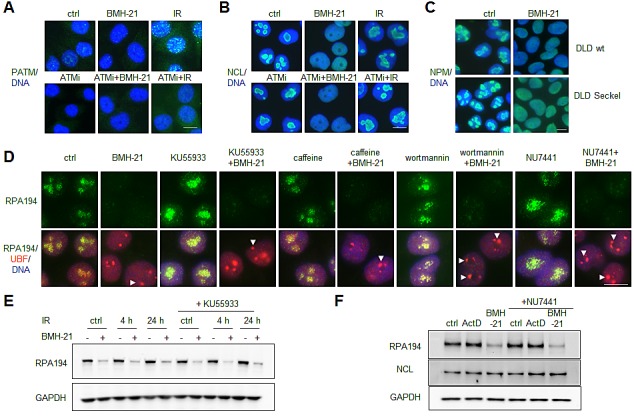
BMH-21 acts in a DNA damage independent manner to activate nucleolar stress and RPA194 degradation (A and B) BMH-21-caused nucleolar stress is independent of ATM pathway activation. A375 cells were pretreated with ATM inhibitor (ATMi) KU55933 (10 μM) for 30 min as indicated, followed by treatment with BMH-21 (1 μM) or IR (2 Gy) and incubation for 3 h. Cells were stained for (A) S1891-phosphorylated ATM (PATM, *green*) or (B) NCL (*green*) and counterstained for DNA (*blue*). (C) Parent DLD and DLD cells with ATR-knock in mutation (DLD Seckel cells) were treated with BMH-21 for 6 h followed by staining for NPM (*green*). Merged images with DNA (*blue*) are shown. (D) Inhibition of DDR pathways does not affect BMH-21-mediated RPA194 degradation. A375 cells were pretreated for 30 min with the following: KU55933 (10 μM), caffeine (2 mM), wortmannin (10 μM), NU7441 (5 μM) followed by addition of BMH-21 (1 μM) and incubation for 2 h. Cells were stained for RPA194 (*green*), UBF (*red*) and counterstained for DNA (*blue*) *Arrowheads*, nucleolar caps. (E) A375 cells were pretreated with KU55933 (10 μM) for 1 h as indicated, followed by IR (2 Gy) and incubation for the indicated times. BMH-21 (1 μM) was added for the final 3 h as indicated. Cell lysates were analyzed by western blotting for RPA194 and GAPDH was used as a loading control. (F) A375 cells were pretreated with NU7441 (10 μM) for 1 h as indicated, followed by addition of ActD (50 ng/ml) or BMH-21 (1 μM) and incubation for 3 h. Cell lysates were analyzed by western blotting for RPA194, NCL and GAPDH was used as a loading control. Scale bars, 10 μm.

ATM is sensitive to alterations in chromatin conformation and DNA damage including those provoked by DNA intercalators. We have earlier shown that BMH-21 does not activate marks of DNA damage, γH2AX or phosphorylation of KAP1, both targets of ATM [13]. To further verify whether BMH-21 impacts ATM activity, we assessed ATM phosphorylation on Ser-1891 (PATM). As controls we used ionizing radiation (IR) to cause ds DNA breaks, and used ATM-specific inhibitor KU55933 to block ATM activity. As shown in Fig. 1A, BMH-21 did not cause ATM phosphorylation. To ask whether BMH-21 activity towards Pol I inhibition depends on ATM kinase activity, we analyzed whether inhibition of ATM activity affects BMH-21-mediated relocalization of nucleolin (NCL), a marker of nucleolar stress. NCL translocation by BMH-21 was prominent also in the presence of abrogated ATM activity (Fig. 1B). Given that BMH-21 causes profound replicative arrest [14] we considered that BMH-21 activity could depend on ATR pathway, the major sensor of replicative stress [6]. To assess this, we used a gene knock-in cell model where the endogenous *ATR* gene has been introduced by mutation of A2101 to G causing ATR inactivation (DLD-Seckel cells, ref. [22]). BMH-21-caused translocation of nucleophosmin (NPM) was intact in these cells (Fig. 1C).

We have shown that degradation of RPA194, the Pol I catalytic subunit, is a unique activity of BMH-21 [14]. To further address whether other key damage signaling and repair pathways could interfere with degradation of RPA194, we pretreated cells with inhibitors of ATM (KU55933), caffeine (ATM/ATR), PI3 kinases (wortmannin) and DNA-PK_cs_ (NU7441), and analyzed the expression and localization of RPA194 and UBF, both markers of active Pol I transcription centers. BMH-21 caused RPA194 degradation and nucleolar cap formation of UBF as we have described before [14], but none of the inhibitors affected these nucleolar responses (Fig. 1D). We further confirmed by western blotting that RPA194 was degraded by BMH-21 in cells with blocked ATM and DNA-PK_cs_ activity (Fig. 1E and). Further, we asked whether DNA damage by IR and activation of DDR could attenuate the efficacy of BMH-21 towards RPA194 degradation. As shown in Fig. 1E, IR pretreatment of the cells 1 or 24 h before addition of BMH-21 (lanes 4 and 6) did not affect RPA194 degradation. We conclude that BMH-21-mediated nucleolar stress and degradation of RPA194 occur independently of DDR and checkpoint activation.

### BMH-21 does not attenuate DNA damage detection

**Figure 2:**
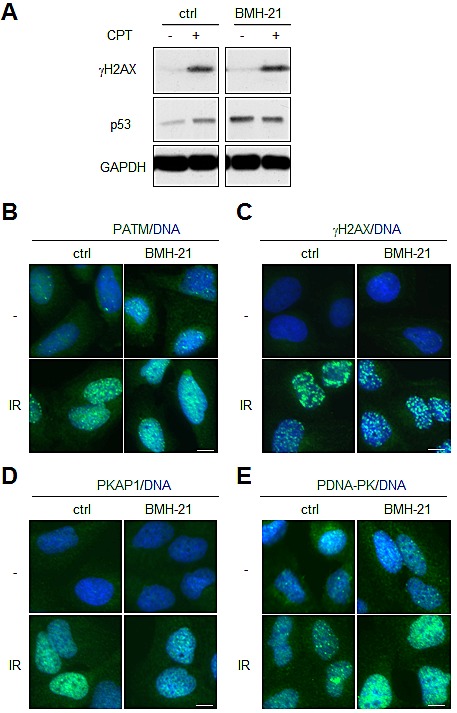
BMH-21 does not protect from activation of DNA damage signaling (A) A375 cells were pretreated with BMH-21 (1 μM) for 2 h followed by addition of camptothecin (CPT 0.5 μM) for 2 h. Cell lysates were analyzed for γH2AX, p53 and GAPDH was used as a loading control. (B-E) U2OS cells were pretreated with BMH-21 (1 μM) for 1 h followed by IR (4 Gy). Cells were fixed and stained for (B) S1891-phosphorylated ATM (PATM), (C) S139-phosphorylated H2AX (γH2AX), (D) S824-phosphorylated KAP1 (PKAP1), (E) S2056-phosphorylated DNA-PK_cs_ (PDNA-PK) and counterstained for DNA (*blue*). Scale bars, 10 μm.

Considering the remarkable lack of engagement of BMH-21 in DDR we considered the possibility that BMH-21 could act to attenuate activated DDR. This could take place by interference with chromatin modeling requisite for damage repair or changes in the nucleosome content [6, 11, 23]. To address this we pretreated cells with camptothecin (CPT) that acts by forming covalent complexes with topoisomerase I and DNA. BMH-21 did not prevent phosphorylation of H2AX caused by CPT (Fig. 2A). Similarly, we treated cells with BMH-21 and IR. BMH-21 co-treatment did not prevent activation of ATM pathway or phosphorylation of its downstream targets H2AX and Ser-824 KAP1 (Fig. 2B-D). In addition, activation of DNA-PK_cs_ as shown by its autophosphorylation on Ser-2056 was not attenuated in the presence of BMH-21 (Fig. 2E). These findings indicate that BMH-21 intercalation with DNA does not prevent the global DDR response activated by DNA breaks.

### Derivatives of BMH-21 convert to DNA damaging modality

**Figure 3:**
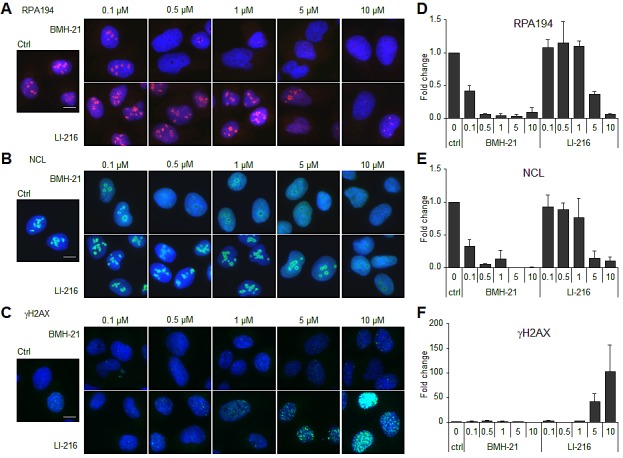
BMH-21 derivative LI-216 activates DNA damage response U2OS cells were treated with the indicated concentrations of BMH-21 and LI-216 for 3 h. Cells were fixed and stained for (A) RPA194, (B) NCL and (C) γH2AX and counterstained for DNA. Scale bars, 10 μm. Image intensities for (D) RPA194, (E) NCL and (F) γH2AX from two independent experiments were quantified, normalized to DNA content and are shown as fold change to control. Bars, mean ± s.e.m.

**Figure 4:**
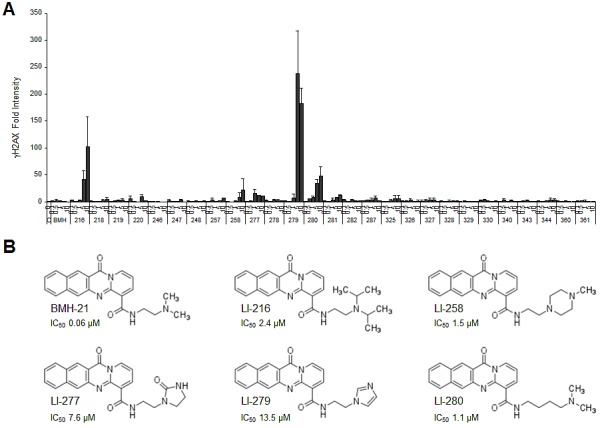
A subset of BMH-21 derivatives activates the DNA damage response (A) U2OS cells were treated with the indicated concentrations of the compounds for 3 h. Cells were fixed and stained for γH2AX and counterstained for DNA. Image intensities from two independent experiments were quantified, normalized to DNA content and are shown as fold change to control. Bars, mean ± s.e.m. (B) Chemical structures of BMH-21 and derivatives that cause DNA damage by 10-fold or more as compared to the control. IC_50_ (μM) indicate inhibition of Pol I activity.

We generated a series of BMH-21 derivatives by altering its *N,N*-dimethylamino carboxamide arm, which we have predicted to interact with the DNA backbone and is critical for BMH-21 activity [14, *manuscript submitted*]. The tetracycle stacking between GC-bases was maintained intact. Given that some derivatives were introduced with moieties that altered the charge and shape of the arm we considered the possibility that these may affect the DNA intercalation cavity, change their DNA interaction modality and could lead to DNA damage. LI-216, where the tertiary basic amine has been substituted with an isopropyl alkyl chain (see Fig. 4B), was first tested for its ability to affect the nucleolar phenotype. As shown in Fig. 3A and B quantified in Fig. 3D and E, LI-216 was over 20 -fold less potent than BMH-21 in causing RPA194 degradation and NCL translocation. However, LI-216 had acquired the ability to cause H2AX phosphorylation at ≥ 5 μM concentration, whereas BMH-21 lacked this ability even at these excessive doses (Fig. 3C and F).

We then subjected the extended series of BMH-21 derivatives to testing for their potency to activate γH2AX responses in cells. In addition to LI-216, compounds LI-258, LI-277, LI-279 and LI-280 caused over 10-fold increase of γH2AX foci formation (Fig. 4A). In contrast, 22 other derivatives were without effect in this regard (Fig. 4A). LI-279 was the most potent activator of DDR by 200-fold increase in γH2AX when the cells were treated at 5-10 μM. All DDR activating derivatives had substantially (20 to 200 –fold) decreased activity to cause nucleolar stress. In the derivatives, the amine had been changed to an imidazole ring (LI-279), oxoimidazolidin (LI-277) or piperazine (LI-258), or the side chain had been extended by two additional carbon linkers (LI-280) (Fig. 4B).

### BMH-21 derivative activates canonical DDR pathways

**Figure 5:**
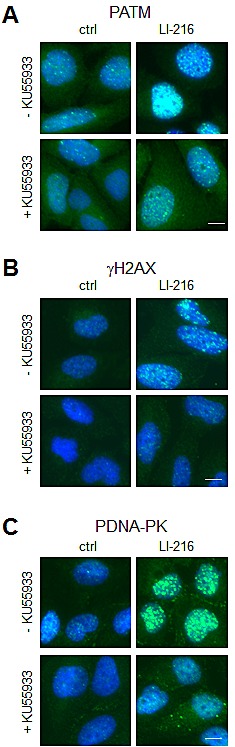
LI-216 activates ATM signaling pathway U2OS cells were treated with LI-216 (10 μM) for 3 h in the presence or absence of KU55933 (10 μM). Cells were fixed and stained for (A) PATM, (B) γH2AX, (C) PDNA-PK and counterstained for DNA. Scale bars, 10 μm.

We then used LI-216 as an example to assess whether the activation of γH2AX conforms to ATM-dependent signaling cascade. Cells were pretreated with KU55933 or not, and were then subjected to LI-216 for 3 hours. Phosphorylation of ATM and H2AX by LI-216 was inhibited by KU55933 and was thus dependent on ATM activity (Fig. 5A and B). Treatment of cells with LI-279 caused similar ATM-dependent DDR response (not shown). These findings are consistent with LI-216 causing ds break-type of DNA damage. Furthermore, DNA-PK_cs_ was phosphorylated in LI-216-treated cells indicating its activation (Fig. 5C).

### DNA damage caused by BMH-21 derivative LI-216 involves NHEJ-dependent repair

**Figure 6:**
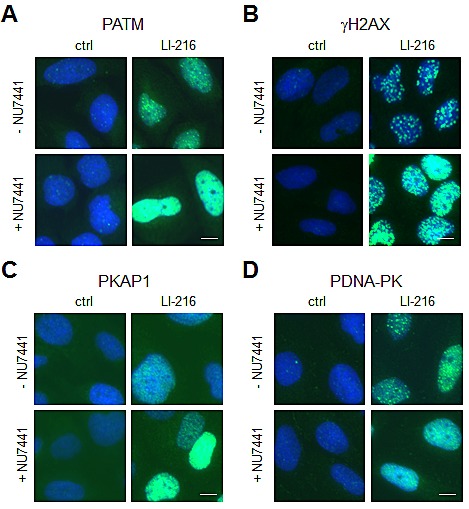
LI-216 mediated DNA damage involves NHEJ-dependent repair U2OS cells were treated with LI-216 (10 μM) for 3 h in the presence or absence of NU7441 (10 μM). Cells were fixed and stained for (A) PATM, (B) γH2AX, (C) PKAP1, (D) PDNA-PK and counterstained for DNA. Scale bars, 10 μm.

As others and us have shown before, inhibition of NHEJ-dependent repair leads to sustained DDR [7, 24, 25]. The engagement of NHEJ following LI-216-caused DNA damage was tested by using DNA-PK_cs_ inhibitor NU7441. Cells were pretreated with NU7441 followed by addition of LI-216, and incubation for 3 hours. Immunostaining for PATM, γH2AX and PKAP1 showed a substantial increase of respective DDR proteins (Fig. 6A-C) and decreased DNA-PK_cs_ phosphorylation consequent to NHEJ-blockade (Fig. 6D). These findings are concordant with that the repair depends on NHEJ.

### Cell viability responses

**Figure 7:**
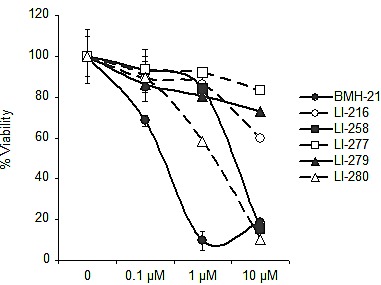
Cell viability assay on selected derivatives U2OS cells were treated with the indicated concentrations of LI-216, LI-258, LI-277, LI-279, LI-280 for 48 h and cell viability was determined using WST-1 assay. Bars, mean ± s.e.m.

To assess whether the acquired property to activate the DNA damage response by the BMH-21 derivatives causes more effective cell killing than BMH-21, we tested the effect of LI-216, LI-258, LI-277, LI-279 and LI-280 on cell viability. Cells were incubated for 48 hours in the presence of the compounds and viability was determined using WST-1 assay. Compounds LI-258 and LI-280 decreased the viability of cells at concentrations that activated the DNA damage response (Fig. 7). However, given that these compounds cause also nucleolar stress at 10 μM concentration, the loss of viability may result from residual inhibition of Pol I, DNA damage or their combination. On the other hand, LI-279 and LI-216, which were the most potent activators of DDR, had no or little effect on cell viability (Fig. 7). We conclude that the loss of viability by the DNA damaging compounds did not exceed that of BMH-21.

## DISCUSSION

DNA intercalation mediates the anticancer activities of widely used chemotherapeutic agents by causing DNA damage and by hindering cellular DNA metabolic processes. We show here that BMH-21, a recently discovered heteroaromatic intercalator, does not activate the cellular DNA damage response and acts independently of the damage signaling and repair pathways to activate markers of nucleolar and Pol I transcription stress. In this regard, BMH-21 differs from other known Pol I inhibitors actinomycin D and CX-5461 by causing the degradation of Pol I catalytic subunit protein RPA194, and by lacking the property to activate DDR. We describe here a set of BMH-21 derivatives and show that the majority of them in which the stacking tetracycle was maintained did not launch DDR. These findings indicate that the heterocycle per se lacks DNA damaging property despite intercalation. However, we also find that five of the derivatives analyzed had an over 10-fold increased DDR response in human cancer cells. These derivatives contain changes in the BMH-21 sidearm that lead to alterations in the sidearm charge and length, and had substantially decreased potencies to cause nucleolar stress. These findings support further development of BMH-21 as a novel class of molecules with the exceptional ability to repress a specified transcriptional process without instigating cellular DNA damage.

We have earlier shown that BMH-21-mediated activation of p53 is independent of cellular DNA damage response as measured by phosphorylation of H2AX and KAP1 and activity of ATM. These findings led us to propose that BMH-21 activities are independent of DDR. Here we studied those responses in respect of the profound activity of BMH-21 to repress Pol I transcription. By using chemical inhibitors of ATM and ATR, the major kinases sensing ss and ds DNA breaks, or ATR-defective cells, we find that neither are required for BMH-21-mediated nucleolar stress response. Furthermore, blocking of DNA-PK_cs_, requisite of NHEJ repair, and which hyperactivates DDR due to accumulation of DNA lesions [24, 25] did not reveal BMH-21-mediation of DDR or attenuated the ability of BMH-21 to target RPA194. These data support and strengthen the notion that inhibition of Pol I transcription by BMH-21 and the associated anticancer activity is independent of DDR.

Molecular modeling of BMH-21 showed that it stacks flatly between GC-bases via π-π intercalation and that its sidearm with the protonated terminal amine assumes a very flat configuration [14]. The tetracycle lies almost parallel with the GC-bases, in contrast to the plane anthraquinone ring of doxorubicin, which is perpendicular to the DNA bases with its side chains protruding to the DNA major and minor grooves [2]. Based on the modeling, BMH-21 does not lead to any significant size exclusion in the major or minor grooves, and is predicted to mostly to cause unwinding of the DNA helix. Given this, DNA damage directed by the derivatives could take place by several not necessarily mutually exclusive mechanisms. These include the protrusion of the side arm into either major or minor grooves, electrophilic addition of DNA bases, free radical interaction with deoxyribose, production of reactive oxygen species, or inhibiting DNA transcription or replication complexes. With this in mind, we have also investigated whether BMH-21 could act as catalytic inhibitor of topoisomerase I or topoisomerase II, without evidence of such activity (ref. [13]). Further molecular modeling and dynamic studies will be needed to reveal BMH-21 interaction modalities with DNA.

Chromatin conformation is an important modulator of DDR [11, 23]. Chromatin compaction and heterochromatinization limits the DDR response, and when heterochromatin is damaged, it is repaired slower than the euchromatin [26]. In addition, DNA intercalator doxorubicin has been shown to cause nucleosome eviction at gene promoters leading to changes in promoter activity or by direct eviction of γH2AX leading to attenuated repair [27, 28]. We hence considered the possibility that BMH-21 intercalation could lead to a global change in the chromatin state that desensitizes the DDR. However, BMH-21 pretreatment attenuated neither the DNA damage caused by IR-induced ds breaks nor by the CPT-type DNA lesions.

Activation of DDR by the DNA damaging derivatives was evident throughout the nucleoplasmic compartment implying that the damage involved genomic DNA lesions. There was no indication of accrual of DDR signals within the nucleolar compartment. Whether repair of rDNA occurs in the nucleolus, in the perinucleolar area or in the nucleoplasm is not known. In yeast, recombinational repair of rDNA has been suggested to take place outside of the nucleolus and is mediated by sumoylation of the Smc5-Smc6 complex, raising the possibility that DNA damage on rDNA could be detected as nucleoplasmic [29].

A major challenge of most chemotherapeutics is normal tissue toxicity. This may result from generation of DNA lesions overwhelming the repair machinery and/or abrogation of essential DNA metabolic processes. Further, many of the intercalating drugs increase the risk of secondary cancers [5]. Given this, much recent effort has been directed towards generation of interventions that provide improved specificity towards cancer cells, DNA target sequences, structures or metabolic processes, or those that exploit synthetic lethality. The absence of DNA damage response of a DNA intercalator that intervenes with a key RNA synthetic cellular process is intriguing and potentially exploitable mechanism of action among cancer chemotherapeutics.

## MATERIALS AND METHODS

### Cells and compounds

The cells were maintained at 37°C in a humidified atmosphere containing 5% CO_2_. A375 were cultured in high-glucose DMEM supplemented with 10% fetal bovine serum (FBS) and U2OS in DMEM supplemented with 15% FBS. 12H-Benzo[g]pyrido[2,1-b]quinazoline-4-carboxamide, N-[2(dimethylamino)ethyl]-12-oxo (BMH-21) was obtained from ChemDiv, verified for purity using LC/MS mass spectrometry and ^1^H-NMR. Other reagents were KU55933 and caffeine (Calbiochem), ActD, camptothecin, wortmannin (Sigma) and NU7441 (Santa Cruz Biotechnology). LI-216, LI-258, LI-277, LI-279, and LI-280 were synthesized from 12-oxo-12H-benzo[g]pyrido[2,1-b]quinazoline-4-carboxylic acid and purified by automated flash chromatography, and verified for purity based on ^1^H NMR (Bruker 400) spectra and analytical LC/MS (Agilent 1260).

### Viability assay

Cells were plated in 96-well plates at a density of 10,000 cells/well and incubated for 48 hours followed by viability measurement using the WST-1 cell proliferation reagent (Roche Diagnostics) according to manufacturer's protocol.

### Immunofluorescence and image analysis

Immunostaining was performed essentially as in ref. [14] and ref. [30]. Cells grown on coverslips were fixed in 3.5% paraformaldehyde, permeabilized with 0.5% NP-40 and blocked in 3% BSA.The following primary antibodies were used: UBF (H-300, Santa Cruz Biotechnology), NCL (4E2, Abcam), RPA194 (C-1, Santa Cruz Biotechnology), phospho-ATM (Cell Signaling Technology), γH2AX (Millipore), phospho-KAP1 (Bethyl Laboratories), phospho-DNA-PKcs (Abcam). Secondary Alexa488 and Alexa594-cojugated anti-mouse and anti-rabbit antibodies were from Invitrogen. DNA was stained with DAPI. Images were captured using Axioplan2 fluorescence microscope (Zeiss) equipped with AxioCam HRc CCD-camera and AxioVision 4.5 software using EC Plan-Neofluar 20x/0.5 and 40x/0.75 objectives (Zeiss). Image analysis was conducted using FrIDA designed for the analysis of RGB color image datasets as in ref. [14] and ref. [25]. Hue saturation and brightness ranges for green and red fluorescence channel and DNA (blue) were defined for each image set. Image intensities were determined as the fraction of positive cells divided total nuclear area as defined by DNA staining. An average of 100 cells was quantified from two fields for each sample.

### Immunoblotting

Cells were lysed in 0.5% NP-40 buffer (25 mM Tris-HCl, pH 8.0, 120 mM NaCl, 0.5% NP-40, 4 mM NaF, 100 μM Na_3_VO_4_, 100 KIU/ml aprotinin, 10 μg/ml leupeptin) or RIPA lysis buffer. Proteins were separated on SDS-PAGE, blotted, probed for respective proteins and detected using ECL (Amersham). The primary antibodies used for detection were NCL (4E2; Abcam), RPA194 (C-1 Santa Cruz Biotechnology). HRP-conjugated secondary antibodies and were from DAKO or Santa Cruz Biotechnology, HRP-conjugated streptavidin was from DAKO.
